# The Effect of Fall Biomechanics on Risk for Hip Fracture in Older Adults: A Cohort Study of Video‐Captured Falls in Long‐Term Care

**DOI:** 10.1002/jbmr.4048

**Published:** 2020-07-06

**Authors:** Yijian Yang, Vicki Komisar, Nataliya Shishov, Bryan Lo, Alexandra MB Korall, Fabio Feldman, Stephen N Robinovitch

**Affiliations:** ^1^ Department of Biomedical Physiology and Kinesiology Simon Fraser University Burnaby BC Canada; ^2^ Department of Sports Science and Physical Education The Chinese University of Hong Kong Hong Kong China; ^3^ George and Fay Yee Centre for Healthcare Innovation University of Manitoba Winnipeg MB Canada; ^4^ Fraser Health Authority Surrey BC Canada; ^5^ School of Engineering Simon Fraser University Burnaby BC Canada

**Keywords:** BIOMECHANICS, FALLS, HIP FRACTURE, HIP PROTECTORS, VIDEO CAPTURE

## Abstract

Over 95% of hip fractures in older adults are caused by falls, yet only 1% to 2% of falls result in hip fracture. Our current understanding of the types of falls that lead to hip fracture is based on reports by the faller or witness. We analyzed videos of real‐life falls in long‐term care to provide objective evidence on the factors that separate falls that result in hip fracture from falls that do not. Between 2007 and 2018, we video‐captured 2377 falls by 646 residents in two long‐term care facilities. Hip fracture was documented in 30 falls. We analyzed each video with a structured questionnaire, and used generalized estimating equations (GEEs) to determine relative risk ratios (RRs) for hip fracture associated with various fall characteristics. All hip fractures involved falls from standing height, and pelvis impact with the ground. After excluding falls from lower than standing height, risk for hip fracture was higher for sideways landing configurations (RR = 5.50; 95% CI, 2.36–12.78) than forward or backward, and for falls causing hip impact (3.38; 95% CI, 1.49–7.67). However, hip fracture risk was just as high in falls initially directed sideways as forward (1.14; 95% CI, 0.49–2.67), due to the tendency for rotation during descent. Falling while using a mobility aid was associated with lower fracture risk (0.30; 95% CI, 0.09–1.00). Seventy percent of hip fractures involved impact to the posterolateral aspect of the pelvis. Hip protectors were worn in 73% of falls, and hip fracture risk was lower in falls where hip protectors were worn (0.45; 95% CI, 0.21–0.99). Age and sex were not associated with fracture risk. There was no evidence of spontaneous fractures. In this first study of video‐captured falls causing hip fracture, we show that the biomechanics of falls involving hip fracture were different than nonfracture falls for fall height, fall direction, impact locations, and use of hip protectors. © 2020 The Authors. *Journal of Bone and Mineral Research* published by American Society for Bone and Mineral Research.

## Introduction

Falls cause 95% of hip fractures in older adults.^1^, ^2^ Nearly 25% of hip fracture patients will die within 1 year of the fracture, and 50% will have major declines in independence.^3^, ^4^ The rates of falls and hip fractures are especially high among older adults in long‐term care (LTC). Only 6% of older adults in Canada live in LTC, but this population experiences 30% of hip fractures.^5^, ^6^ Strategies that are effective in preventing falls in community‐dwelling seniors (eg, exercise) have been unsuccessful in LTC, due to the high prevalence of physical and cognitive impairment in residents.^7^ This highlights the importance of complementary strategies in LTC for preventing hip fracture in the event of a fall, through approaches such as vitamin D and calcium supplementation, pharmacologic therapy, and the use of wearable hip protectors,^5^, ^8^ which may be especially beneficial for individuals with low body mass index (BMI) who have higher risk for fracture.^9^, ^10^, ^11^ Improved understanding of the factors that separate injurious and noninjurious falls may lead to refinements to existing strategies, and new approaches to prevention.

Previous case‐control studies in older adults have found that risk for hip fracture in a fall depends at least as much on the biomechanics of the fall, as it does on bone density.^12^, ^39^ In particular, although a one standard deviation (1SD) decline in bone density increased fracture risk twofold to threefold,^12^ falling sideways increased fracture risk sixfold,^13^ and landing on the hip increased fracture risk 30‐fold.^14^ Furthermore, hip fractures were less common when the person landed on their hand, or grabbed or hit an object to break the fall.^39^ However, a major limitation of these studies is their reliance on interviews or questionnaires completed by the faller and witnesses (if any) to determine a narrow range of fall characteristics (fall direction, fall height, and contact sites). Recalling the circumstances of falls is challenging, especially for older adults with cognitive impairment.^15^, ^16^ Also, fallers who sustained a fracture may bias their response based on the notion that they must have landed on the hip for fracture to occur.^11^ To date, objective evidence on the circumstances of falls causing hip fracture has not been available to overcome these limitations.

In this study, we address this knowledge gap by analyzing videos of real‐life falls experienced by older adults living in LTC, and comparing the characteristics of falls that did result in hip fracture versus those that did not result in hip fracture. We hypothesized that fracture risk would associate with fall characteristics that have previously been shown to be important, as reviewed above (fall height, fall direction, contact sites, use of hip protectors, and BMI). We considered initial fall direction and landing configuration separately, based on our previous observation that falls in LTC often involve rotation during descent.^17^ In addition to hip and hand impact, we examined knee impact (which may decrease impact severity to the hip), use of mobility aids, and attempts to recover balance by stepping, which we previously found to decrease hip impact velocity.^18^ We also tested whether fracture risk, as hypothesized by Cummings and Nevitt,^19^ associated with activity at the time of the fall (eg, walking versus standing), and with cause of imbalance.

## Subjects and Methods

### Participants and setting

**Fig 1:**
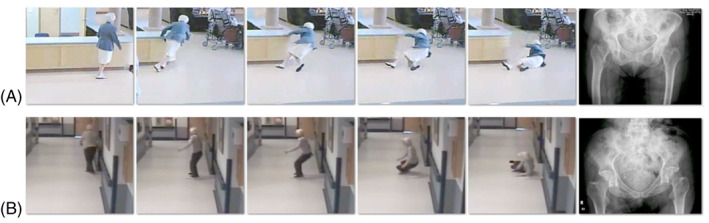
Snapshots from video footage of falls resulting in hip fracture. (*A*) This 94‐year‐old woman experienced a right‐side intertrochanteric hip fracture, from falling due a trip during walking. She fell forward but rotated during descent to impact the right side of her pelvis. (*B*) This 84‐year‐old woman experienced a left‐side femoral neck fracture after losing balance while standing and turning. She fell sideways and landed sideways, impacting the left side of her pelvis. Videos of falls (with and without hip fracture) are included in the Supplementary Material.

We collected video footage of 2377 falls experienced by 646 residents (mean ± SD, age = 82.6 ± 9.1 years; 57% female) of two LTC facilities (Delta View Life Enrichment Centre, Delta, BC, Canada, and New Vista Society Care Home, Burnaby, BC, Canada) affiliated with the Fraser Health Authority in British Columbia (see snapshots of samples falls causing hip fracture in Fig. 1, and Supplementary Material for videos of falls with and without hip fracture). A fall was defined as an unexpected event during which the person comes to rest on the floor or other lower level, with or without an injury.^20^ All falls took place from April 2007 to March 2018. All cameras (*n* = 216 and *n* = 48 at Delta View and New Vista, respectively) were located in common areas (eg, dining rooms, lounges, hallways). Videos were acquired and stored at a recording rate of 15 to 30 frames/s, with a minimal resolution of 640 × 480 pixels.

For each fall, we also collected data from incident reports completed by nursing staff, which documented the nature and location of injuries from the fall, and whether or not the resident was wearing a hip protector at the time of the fall. Incident reports also contained data on resident age, sex, height, weight, and disease diagnoses. The accuracy of incident report data was confirmed through review of medical records for the 7‐day period after the fall. In all cases where hip fracture was noted (*n* = 30), the diagnosis was confirmed through International Classification of Diseases, Ninth Revision (ICD‐9) codes from hospital records.

The research ethics board at Simon Fraser University approved this study. Upon admission to the LTC facility, each resident or proxy provided permission for the facility to acquire video footage in common areas for safety purposes. Video footage of falls, along with clinical incident reports, were shared with the research team as secondary data. A portion of residents (*n* = 302) provided separate written informed consent for access to medical records, and for sharing of video and images for research and education.

### Data analysis

Each fall video was analyzed by a team of three trained evaluators (blinded to the participant's fracture status), using a validated questionnaire^21^ to classify observable characteristics of the initiation, descent and impact stage of the fall. Although most studies have classified falls as having a single falling direction, we recently reported, based on video analysis, the common tendency for residents of LTC to rotate during descent to change the fall direction.^17^, ^22^ Accordingly, we separately identified and analyzed how fracture risk depends on the initial fall direction (at the onset of imbalance), and the landing configuration. Our explanatory variables included: (i) fall height (classified as lower than standing height, versus standing height or higher); (ii) cause of imbalance (classified as intrinsic [eg, incorrect weight shift, loss of support, collapse] or extrinsic [eg, trip, bump, slip]); (iii) activity at the time of the fall (walking, standing, or transferring); (iv) use of mobility aids (eg, walkers) at the time of falling (yes or no); (v) initial fall direction (predominantly forward, sideways, backward, or straight down); (vi) landing configuration (predominantly forward, sideways, or backward); (vii) body rotation during descent (reflecting a change from initial fall direction to landing configuration; classified as none, forward rotation, or backward rotation); (viii) occurrence of impact to the hip region of the pelvis (left or right hip/buttock; classified as yes or no); (ix) occurrence of attempts to recover balance by stepping, or reach‐to‐grasp of a nearby support (yes or no); (x) occurrence of attempts to arrest the fall with the outstretched hand(s) (yes or no); (xi) impact to the knee(s) (yes or no); and (xii) hip protector use (yes or no). Hip protector use was derived from fall incident reports. Although Safehip and Hipsaver were the two most commonly used types of hip protectors, incident reports did not distinguish which product was being used. All other explanatory variables were derived from video analysis.

For falls involving hip fracture, we conducted additional analysis to determine the temporal sequence of impacts to body parts, and the orientation of the pelvis when it first impacted the ground. Pelvis orientation was classified in 30‐degree increments, ranging from 0 degrees (impact to the posterior aspect of the pelvis, with both buttocks contacting the ground) to 180 degrees (impact to the anterior aspect of the pelvis).

### Statistics

We examined associations between risk for hip fracture and each explanatory variable using generalized estimating equation (GEE) models, which account for potential correlation between repeated falls in a given individual. We performed negative binomial regression (with log link) to calculate the relative risk ratio of hip fracture for each explanatory variable. Age (continuous) and sex (dichotomous) were included as covariates in each model, given their known influence on risk for hip fracture.^23^, ^24^, ^25^ For each primary variable, we report *p* values, the relative risk (RR), and corresponding 95% confidence interval (CI) between falls with and without hip fracture. All statistical analyses were conducted on SPSS 24 (IBM Corp., Armonk, NY, USA). The significance level was set at *α* = 0.05.

## Results

### Hip fractures

**Fig 2:**
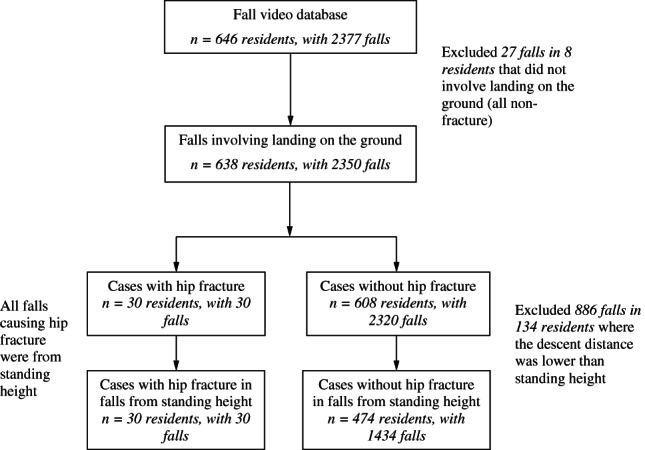
Flowchart of sample selection (participants and falls) in this study.

Of the 2377 video‐captured falls that we examined, 30 falls (1.26%) resulted in hip fracture (Fig. 2) confirmed by ICD‐9 codes. Of the six hip fractures where we had access to radiographs, five were femoral neck fractures, and one was an intertrochanteric fracture.

### Fall height

From video analysis, we observed that 100% of hip fracture cases involved falls from standing height, whereas for nonfracture falls, 61.8% were from standing height, 37.9% were from lower than standing height (eg, falling while sitting), and 0.2% (*n* = 4) were from greater than standing height. The portion of falls from standing height was greater in falls causing hip fracture than in non‐fracture control falls (*p* < .001 based on Fisher's exact test). We also found that the pelvis impacted the ground in 100% of falls causing hip fracture, and 90.5% of nonfracture falls (*p* = .5). For all subsequent comparisons, we excluded nonfracture falls where no body part contacted the ground (eg, falls into chairs; *n* = 27 in eight residents), and falls from lower than standing height (*n* = 886 in 134 residents), resulting in a final nonfracture (control) group of 1434 falls in 474 residents (Fig. 2).

### Resident characteristics

There were no significant differences between fracture and nonfracture groups in sex, height, BMI, or disease diagnoses (Table 1). There were nonsignificant trends for residents who experienced hip fracture being older (*p* = .085), having lower body weight (*p* = .065), and having diagnosed hypertension (*p* = .052), or osteoporosis (*p* = .070), when compared to participants who fell but did not experience fracture. As covariates in our GEE models, age and sex were not associated with risk for hip fracture (*p* > .05). The average number of video‐captured falls per participant was 4.1 for persons with hip fracture, and 3.7 for those without. This difference was not statistically significant.

**Table 1:** Characteristics of Residents and Baseline Characteristics of Falls from Video Analysis

Characteristic	Falls causing hip fracture (*n* = 30 residents, 30 falls)	Falls not causing hip fracture (*n* = 409 residents, 1292 falls)^a^	*p* ^b^
Demographics			
Age (years), mean ± SD	85.4 ± 7.9	82.3 ± 10.0	0.085
Female, *n* (%)	19 (63.3)	233 (57.0)	0.569
Anthropometry			
Height (cm), mean ± SD	162.0 ± 11.6	163.9 ± 10.8	0.389
Weight (kg), mean ± SD	60.0 ± 13.7	66.1 ± 16.2	0.065
BMI (kg/m^2^), mean ± SD	23.0 ± 5.4	24.4 ± 4.7	0.155
Disease diagnoses, *n* (%)			
Alzheimer's disease	8 (26.7)	103 (25.2)	0.830
Dementia	23 (76.7)	261 (63.8)	0.172
Cardiac arrhythmia	0 (0.0)	30 (7.3)	0.250
Congestive heart failure	4 (13.3)	34 (8.3)	0.315
Hypertension	17 (56.7)	155 (37.9)	0.052
Stroke	4 (13.3)	64 (15.6)	1.000
Diabetes mellitus	5 (16.7)	97 (23.7)	0.503
Osteoporosis	9 (30.0)	64 (15.6)	0.070
Parkinson's disease	0 (0.00)	23 (5.6)	0.390
COPD	3 (10.0)	45 (11.0)	1.000

BMI = body mass index; COPD = chronic obstructive pulmonary disease; SD = standard deviation.

a Among the 474 nonfracture residents, we could confirm disease diagnoses for only 409 (with 1292 falls).

b Comparisons were based on Fisher's exact tests (categorical variables) or *t* tests (continuous variables).

### Cause and activity at time of falling

There were no differences between fracture and nonfracture falls in the cause of the fall (extrinsic versus intrinsic), and activity at the time of falling (walking versus standing or transferring; Table 2). Use of a mobility aid at the time of falling reduced fracture risk (RR 0.30; 95% CI, 0.09–1.00). Among the 30 fracture cases, there were no obvious signs of pain, or the leg giving away prior to the fall, which might indicate a “spontaneous” fracture occurring before the fall.

**Table 2:** Associations Between Hip Fracture Risk and the Characteristics of Falls From Standing Height (*n* = 1464)

	Falls *n* (%)		
Characteristic	Hip fracture (*n* = 30; 30 falls)	No hip fracture (*n* = 474; 1434 falls)	RR (95% CI)^a^
Fall initiation stage			
Extrinsic cause of imbalance	11 (36.7)	510 (35.7)	1.11 (0.52–2.40)
Versus intrinsic cause	19 (63.3)	919 (64.3)	1
Walking	14 (46.7)	801 (55.9)	0.68 (0.33–1.41)
Versus standing or transferring	16 (53.3)	631 (44.1)	1
Using a mobility aid	3 (10.0)	347(24.3)	0.30 (0.09–1.00)*
Versus none	27 (90.0)	1082 (75.7)	1
Initially falling sideways	17 (56.7)	515 (35.9)	4.62 (1.65–12.95)*
Initially falling forward	9 (30.0)	328 (22.9)	4.02 (1.22–13.26)*
Versus backwards or straight down	4 (13.3)	590 (41.2)	1
Fall descent stage			
Stepping response	25 (83.3)	1031 (72.0)	1.87 (0.72–4.65)
Versus none	5 (16.7)	400 (28.0)	1
Reaching to grasp	7 (23.3)	206 (14.9)	1.74 (0.68–4.46)
Versus none	23 (76.7)	1177 (85.1)	1
Fall impact stage			
Landing sideways	23 (76.7)	531 (37.1)	5.50 (2.36–12.78)*
Versus landing forward or backward	7 (23.3)	899 (62.9)	1
Hip impact	22 (73.3)	644 (45.0)	3.38 (1.49–7.67)*
Versus no hip impact	8 (26.7)	786 (55.0)	1
Hand(s) arrest^b^	18 (69.2)	850 (81.3)	0.58 (0.25–1.36)
Versus no hand(s) arrest	8 (30.8)	195 (18.7)	1
Knee impact	20 (66.7)	670 (47.0)	2.27 (1.12–4.63)*
Versus no knee impact	10 (33.3)	757 (53.0)	1
Wearing hip protector^c^			
Yes	16 (57.1)	927 (73.5)	0.45 (0.21–0.99)*
Versus no	12 (42.9)	335 (26.5)	1

As a covariate in all models, age and sex were not significantly associated with the risk of hip fracture.

GEE = generalized estimating equation; RR = relative risk ratio.

a Comparisons were based on GEE models.

b Defined as hand contact with the environment that was perceived as deliberate.

c Use of hip protector was documented in 1290 falls, including 28 hip fracture cases.

* Statistically significant (*p* ≤ .05).

### Fall direction

Risk for hip fracture was associated with initial fall direction, landing direction, and body rotation during descent (Tables 2 and 3). Among falls from standing height, hip fracture risk was higher in falls initially directed sideways or forward, versus backwards or straight down (RR 4.62; 95% CI, 1.65–12.95 for sideways and RR 4.02; 95% CI, 1.22–13.26 for forward). Risk for hip fracture was not different for falls initially directed sideways versus forward (RR 1.14; 95% CI, 0.49–2.67). However, landing sideways created higher risk for hip fracture, when compared to other landing directions (RR 5.50; 95% CI, 2.36–12.78). In falls that were initially directed backward, rotating to land sideways (observed in 22% of cases) increased hip fracture risk (RR 10.31; 95% CI, 1.09–100.0). In falls that were initially directed sideways, rotating to land backward (which occurred in 42% of cases) reduced fracture risk (RR 0.28; 95% CI, 0.08–0.96). Although rotation from sideways to forward was less common (occurring in 5.1% of cases; *n* = 26), none of these falls resulted in hip fracture.

**Table 3:** Association Between Hip Fracture Risk and Body Rotation During Descent Among Falls From Standing Height (Combinations of Initial Fall Direction and Landing Configuration)

Change of fall direction		Falls *n* (%)			
Initial fall direction	Landing configuration	Hip fracture	No hip fracture	RR (95% CI)	*p*
Forward (*n* = 337)					
	Forward	1 (11.1)	134 (40.9)	1	
	Backward	2 (22.2)	59 (18.0)	4.43 (0.41–48.21)	0.222
	Sideways	6 (66.7)	135 (41.2)	5.75 (0.70–47.41)	0.104
Backward (*n* = 500)					
	Forward	0 (0)	3 (0.6)	^a^	
	Backward	1 (25.0)	384 (77.4)	1	
	Sideways	3 (75.0)	109 (22.0)	10.31 (1.09–100.0)	0.042*
Sideways (*n* = 530)					
	Forward	0 (0)	26 (5.1)	^a^	
	Backward	3 (18.8)	216 (42.1)	0.28 (0.08–0.96)	0.044*
	Sideways	14 (82.4)	271 (52.8)	1	
Straight down (*n* = 68)					
	Forward	0 (0)	9 (9.7)	^a^	
	Backward	0 (0)	68 (73.1)	^a^	
	Sideways	0 (0)	16 (17.2)	^a^	

RR = relative risk ratio.

a We were unable to calculate an RR owing to 0 cases in the hip fracture group.

* Statistically significant (*p* ≤ .05).

### Hip impact

**Fig 3:**
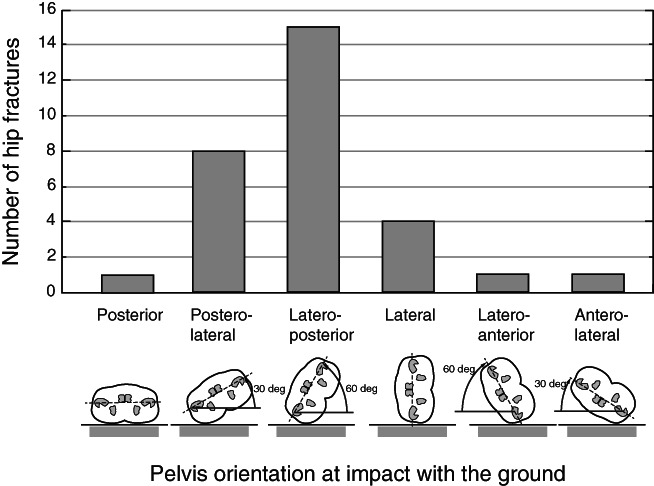
Distribution of pelvis orientation at impact with the ground for hip fracture cases (*n* = 30).

Impact to the hip increased the risk for hip fracture (RR 3.38; 95% CI, 1.49–7.67). From our additional analysis of hip fracture cases (Fig. 3), we found that 23 of 30 cases (77%) involved impact to the posterolateral aspect of the pelvis, oriented either at 60 degrees (15 cases) or 30 degrees (eight cases) from the ground, four of 30 (13%) involved impact to the lateral aspect of the pelvis, two of 30 (7%) involved impact to the anterolateral aspect of the pelvis, and one of 30 (3%) involved impact to the posterior aspect of the pelvis. Aside from the one case involving impact to the posterior aspect of the pelvis, all hip fractures involved impact to the pelvis on the fracture side.

### Upper limb and knee contact

There were no differences between fracture and nonfracture groups in use of the upper limbs to arrest the fall (RR 0.58; 95% CI, 0.25–1.36), reaching to contact or grasp a nearby support (RR 1.74; 95% CI, 0.68–4.46), or attempts to recover balance by stepping (RR 1.87; 95% CI, 0.72–4.65). The occurrence of knee impact was associated with higher risk for hip fracture (RR 2.27; 95% CI, 1.12–4.63). In additional analysis of hip fracture cases, we characterized the sequence of impacts to the first three body segments contacting the ground (Supplementary Table S1). In all cases, the pelvis was among the first three body parts to impact the ground. The pelvis was the first body segment to impact the ground in five of 30 fractures (17%), the second in 12 of 30 (40%) of cases, and the third in 13 of 30 (43%) of cases. Upper‐limb (hand or elbow) impact preceded pelvis impact in 20 of 30 (67%) of cases, and occurred simultaneously with pelvis impact in two of 30 (7%) of cases. Knee impact preceded pelvis impact in 18 of 30 (60%) of hip fractures.

### Hip protector use

Hip protector use reduced the risk for hip fracture (RR 0.45; 95% CI, 0.21–0.99). Hip protectors were worn in 73% of falls without hip fracture, and in 57% of falls with hip fracture. Of the 16 hip fractures that occurred while wearing hip protectors, the aspect of the pelvis that impacted the ground was posterior in one case (6%), posterolateral in 12 cases (75%), lateral in two (13%), and anterolateral in one (6%). These trends are similar to those observed for all fractures (Fig. 3).

## Discussion

We analyzed video footage of falls experienced by older adults in LTC, to provide the first objective evidence of how the biomechanics of falls affect risk for hip fracture in this high‐risk population. Our approach overcomes the questionable accuracy of self‐reported fall characteristics,^15^, ^16^ and allowed us to examine fall characteristics in greater detail (eg, body rotation during descent, pelvis impact configurations) than research published previously. We found that the biomechanics of falls in LTC involving hip fracture were different than nonfracture falls for fall height, fall direction, impact locations, and use of hip protectors.

We found that fracture risk was higher in falls from standing height or higher, than in falls from lower than standing height. All hip fractures were caused by falls from standing height, whereas 38% of nonfracture falls occurred from lower than standing height (eg, falling while sitting). This finding agrees with results from previous studies,^1^, ^12^, ^26^ including Grisso and colleagues,^27^ who found that falls from standing height created 2.4‐fold greater odds for hip fracture than falls from lower heights.

We observed no differences between the fracture and nonfracture groups in BMI, body height, and body weight, although there was a trend (*p* = .065) toward increased fracture risk with decreasing body weight. Previous studies of fracture risk in LTC residents^11^ and among community‐dwelling women^10^ found that BMI and body weight were negatively associated with fracture risk. For example, Compston and colleagues^10^ reported mean differences between fracture and nonfracture groups of 0.3 kg/m^2^ for BMI and 0.9 kg for body weight. In our study, the (nonsignificant) differences between fracture and nonfracture groups were 1.4 kg/m^2^ and 5.6 kg. Our sample size may have been too small to detect the effect of BMI on fracture risk. Alternatively, the high use of hip protectors in the LTC homes that we studied may have lessened the effect of low BMI on fracture risk, by supplementing the natural padding provided by trochanteric soft tissues.^28^

We found that 38% of falls involved body rotation during descent, to change the primary direction of the fall from initiation to landing. The high prevalence of rotation is similar to that documented in our previous results,^17^, ^22^ and highlights the importance of separately examining how fracture risk depends on the initial fall direction (at the onset of imbalance), and the landing configuration.

With regard to initial fall direction, we found that fracture risk was just as high in falls initially directed forward as sideways. When compared to falls initially directed backward or straight down, both forward and sideways falls created similar increases in risk (between fourfold to fivefold). However, for landing configuration, fracture risk was 5.5‐fold greater in falls with sideways landings than in falls with forward or backward landings. The discrepancy is explained by the observation that, in 42% of falls that were initially directed forward, individuals rotated to land sideways. Conversely, in 41% of falls that were initially directed sideways, individuals rotated to land backward, and this reduced fracture risk 3.6‐fold when compared to no rotation. Although rotation from backward to sideways was less common (occurring in 22% of falls initially directed backward), fracture risk was 10‐fold greater in these falls, than in backward falls with no rotation. The notion that older adults can modify sideways falls to land backward, and thereby reduce their risk for hip fracture, deserves further consideration as a potential target for exercise‐based prevention strategies.

Although previous studies did not specify whether they defined “fall direction” as the initial fall direction or landing configuration, our results for landing configuration are in agreement with those reported by Greenspan and colleagues,^13^ who found that sideways falls had 5.7‐fold higher odds for hip fracture than falls in other directions; by Nevitt and Cummings,^39^ who reported that sideways or straight‐down falls had 3.3‐fold higher odds for fracture; and by Hwang and colleagues,^29^ who found that, when compared to forward falls, both backward and sideways falls created over 10‐fold higher risk for hip fracture. Future studies should separately consider and report outcomes for initial fall direction and landing direction.

Our results show that individuals in LTC exhibit a clear tendency to rotate during the descent stage of falls, to land sideways in falls that were initially directed forward, and to land backward in falls initially directed sideways. The question arising is: What might be the rationale for this behavior? The answer may relate to the priority of protecting the head while falling. We previously found that the odds for head impact from falls in LTC were 3.5‐fold higher in forward landings than in sideways or backward landings (perhaps secondary to age‐related declines in the ability to effectively arrest a forward fall with the upper limbs, which is a strength‐demanding task^30^). We also found that rotating from forward to land sideways reduced the odds for head impact 2.8‐fold.^22^ Further research is required to determine whether a primary factor underlying the tendency to rotate during forward falls is loss of upper limb strength—a modifiable risk factor.

In 29 of 30 fractures, impact occurred to the pelvis on the fracture side, and hip fractures were six times more likely to involve impact to the posterolateral aspect of the pelvis than the lateral aspect. This observation is consistent with results from finite element modeling, which found that the fracture strength of the proximal femur is lowest for impact forces that are directed posterolaterally,^31^ and from laboratory experiments, showing that posterolateral falls result in greater contact force.^32^

Risk for hip fracture was threefold lower in cases where individuals fell while holding a mobility aid, which was observed in 24% of nonfracture falls and 10% of falls causing hip fracture. The mobility aid was a walker or rollator in 75% of cases, a wheelchair in 22% of cases, and a cane in 3% of cases. The reduction in fracture risk may relate to the ability of individuals to exert downward forces through hand contact with the aid, to slow descent and reduce the severity of the impact.

In contrast, arresting the fall with the outstretched hand(s), which occurred in 81% of cases, and successful reach‐to‐grasp responses, which occurred in 15% of falls, did not associate with fracture risk. The nature of these responses varied widely, and the occurrence of upper limb contact provides little indication of the force generation and energy absorption involved in the impact. We previously reported that hand impact did not affect the risk for head impact during falls by residents of LTC.^22^, ^33^ Others have reported that arresting the fall with the hands associated with reduced risk for hip fracture among community‐dwelling individuals,^14^, ^39^ but had no effect on hip fracture risk for older adults residing in nursing homes.^13^ The discrepancy may be due to differences between LTC and community‐based populations in factors such as upper limb strength and flexibility.

We found that hip protectors, which were worn in 73% of falls, reduced hip fracture risk 2.2‐fold. These trends agree with results from our recent analysis of 3520 falls occurring over 12 months in 14 LTC facilities in Fraser Health, where residents wore hip protectors in 60% of falls, and hip protectors provided a threefold reduction in the risk for hip fracture.^34^ According to a Cochrane meta‐analysis, hip fracture risk is reduced by 18% (RR 0.82; 95% CI, 0.67–1.00) when LTC residents are offered hip protectors.^8^ However, these estimates were derived from an “intention to treat” analysis of trials where adherence in wearing hip protectors often fell below 50%, and where the types (and protective value) of hip protectors varied across trials. In contrast, our results reflect the value of specific types of hip protectors (Safehip and Hipsaver) for residents of LTC who are willing to wear them. Our finding that 77% of hip fractures occurred from impacts to the posterolateral aspect of the hip may help to guide improvements in the design of hip protectors, which are currently designed to protect against lateral impacts.^35^, ^36^

Our observed rate of hip fracture is within the range reported previously for older adults in LTC. Of the 2377 falls we captured and analyzed, 1.26% resulted in hip fracture. Based on review of 12 studies, Rubenstein and colleagues^37^ reported that 4% of falls in LTC resulted in any type of facture. Recent studies of falls and fractures in LTC homes in the Fraser Health region of British Columbia (which reported rates of adherence in wearing hip protectors that were similar to ours) found that 0.6% of falls caused hip fractures,^34^ and 0.9% of falls resulted in any type of fracture.^38^ In contrast, Nevitt and Cummings^13^ found that only 0.2% of falls experienced by adults aged 60 years or older living in the community caused hip fracture. Because our analysis focused on falls in common areas captured on video (and did not include falls and fractures in private areas), we cannot determine exact fracture rates per person‐year. However, we previously described that residents experienced a median of 2.5 falls/year, based on analysis of all falls (in both common and private areas) in a subset of 220 residents in our cohort.^40^ Based on our current finding of 1.26% of falls causing hip fracture (and assuming this rate applies to falls in private as well as common areas), this would translate to a fracture rate of 31.5 hip fractures/1000 person‐years. This fracture rate is comparable to reports of 68.6 fractures/1000 person‐years for women and 49.8 fractures/1000 person‐years for men in Canadian LTC homes,^41^ 41.0 fractures/1000 person‐years in LTC homes in Germany,^42^ and 23 fractures/1000 person‐year in LTC homes in the United States.^43^

Our study had important limitations. First, we analyzed video footage of falls experienced by older adults in LTC, which occurred in common areas of the facilities (dining rooms, lounges, and hallways). Accordingly, our results may not apply to falls in private areas (bedrooms or bathrooms), or to falls on stairs, or to falls experienced by healthier older adults living in the community. Second, we focused our analysis on biomechanical risk factors for hip fracture, and although we were able to compare disease diagnoses from fall incident reports, the small sample of participants with hip fractures who provided us with consent to access medical records prevented us from examining how fracture risk associated with factors such as bone density, muscle strength, cognitive status, or medication use. Third, we did not examine how hip fracture risk depends on markers of impact severity such as the contact velocity of the pelvis, which merits exploration in future work.

In summary, our results show that risk for hip fracture in LTC was just as high in falls that were initially directed forward, as in falls that were initially directed sideways. These trends are explained by the common tendency to rotate from forward to sideways during descent. Future studies should recognize that many falls (nearly 40% in our study) cannot be adequately described as having a single fall direction, and separately consider fall direction at initiation versus landing. We also show that protective responses that have been found to be effective in community‐dwelling older adults, such as bracing the fall with the upper limbs,^39^ did not associate with fracture risk among LTC residents, perhaps due to insufficient upper limb force generation and energy absorption. Finally, our results show the value of hip protectors for reducing the risk for hip fracture among LTC residents, and suggest improvements in the design of hip protectors to protect against impact to the posterolateral aspect of the hip, which accounted for 77% of hip fractures.

## Disclosures

FF has been a consultant on biomechanical testing of hip protectors for Blue Tree Medical. SNR has been a consultant on biomechanical testing of hip protectors for Tytex and Blue Tree Medical. YY, VK, NS, BL, and AMBK state that they have no conflicts of interest.

## Peer Review

The peer review history for this article is available at https://publons.com/publon/10.1002/jbmr.4048.

## Supporting information


**Supplementary Table S1** Temporal sequence of impacts to body sites in falls causing hip fracture (*n* = 30).

**Supplementary Material** Videos of real‐life falls in older adults.
