# Supplementary material for: The Effect of Fall Biomechanics on Risk for Hip Fracture in Older Adults: A Cohort Study of Video‐Captured Falls in Long‐Term Care
Source: J Bone Miner Res. 2020 Jul 6;35(10):1914–22. doi: 10.1002/jbmr.4048 (PMC7689902; doi:10.1002/jbmr.4048)
Supplement: Supplementary file 1 — Supplementary Table S1 Temporal sequence of impacts to body sites in falls causing hip fracture (n = 30). [file JBMR-35-1914-s001.docx]

**Supplementary Table S1.** Temporal sequence of impacts to body sites in falls causing hip fracture (n=30).

| **Sequence of impact during the fall** | | | **Number (percent)** |
| --- | --- | --- | --- |
| **First impact** | **Second impact** | **Third impact** |  |
| Posterolateral pelvis | Elbow | Wrist | 1 (3.3%) |
| Posterolateral pelvis | Wrist/knee | Elbow | 1 (3.3%) |
| Posterior pelvis/wrist | Elbow(s) | Head | 2 (6.7%) |
| Posterolateral pelvis/wrist | Elbow | Torso | 1 (3.3%) |
| Wrist | Posterolateral pelvis | Elbow | 3 (10%) |
| Wrist | Lateral pelvis | Elbow | 1 (3.3%) |
| Knee/wrist | Posterolateral pelvis | Torso | 2 (6.7%) |
| Wrist/knee | Anterolateral pelvis | Wrist/elbow | 1 (3.3%) |
| Knee | Lateral pelvis | Torso | 1 (3.3%) |
| Knee | Posterolateral pelvis | Wrist/knee | 2 (6.7%) |
| Knee | Posterior pelvis | Torso | 1 (3.3%) |
| Shoulder | Posterolateral pelvis | Wrist | 1 (3.3%) |
| Wrist | Knee | Posterolateral pelvis | 4 (13.3%) |
| Wrist(s) | Elbow(s) | Posterolateral pelvis | 2 (6.7%) |
| Knee(s) | Wrist(s) | Lateral pelvis | 3 (10%) |
| Knee(s) | Wrist(s) | Posterolateral pelvis | 2 (6.7%) |
| Knee | Wrist | Anterolateral pelvis | 1 (3.3%) |
| Knee | Elbow | Posterolateral pelvis | 1 (3.3%) |
